# *Sanghuangporus vaninii* extract ameliorates hyperlipidemia in rats by mechanisms identified with transcriptome analysis

**DOI:** 10.1002/fsn3.4002

**Published:** 2024-02-16

**Authors:** Ning Gao, Yuanzhen Liu, Guangjie Liu, Bo Liu, Yupeng Cheng

**Affiliations:** ^1^ Key Laboratory of Basic and Application Research of Beiyao (Heilongjiang University of Chinese Medicine), Ministry of Education Harbin China; ^2^ School of Pharmacy Heilongjiang University of Chinese Medicine Harbin China; ^3^ School of Pharmaceutical Engineering Heilongjiang Agricultural Reclamation Vocational College Harbin China

**Keywords:** edible and medicinal fungi, hyperlipidemia, RNA‐seq analysis, *Sanghuangporus vaninii*

## Abstract

The increasing incidence of hyperlipidemia is a serious threat to public health. The development of effective and safe lipid‐lowering drugs with few side effects is necessary. The purpose of this study was to assess the lipid‐lowering activity of *Sanghuangporus vaninii* extract (SVE) in rat experiments and reveal the molecular mechanism by transcriptome analysis. Hyperlipidemia was induced in the animals using a high‐fat diet for 4 weeks. At the end of the 4th week, hyperlipidemic rats were assigned into two control groups (model and positive simvastatin control) and three treatment groups that received SVE at 200, 400, or 800 mg kg^−1^ day^−1^ for another 4 weeks. A last control group comprised normal chow‐fed rats. At the end of the 8th week, rats were sacrificed and lipid serum levels, histopathology, and liver transcriptome profiles were determined. SVE was demonstrated to relieve the lipid disorder and improve histopathological liver changes in a dose‐dependent manner. The transcriptomic analysis identified changes in hepatocyte gene activity for major pathways including steroid biosynthesis, bile secretion, cholesterol metabolism, AMPK signaling, thyroid hormone signaling, and glucagon signaling. The changed expression of crucial genes in the different groups was confirmed by qPCR. Collectively, this study revealed that SVE could relieve hyperlipidemia in rats, the molecular mechanism might be to promote the metabolism of lipids and the excretion of cholesterol, inhibit the biosynthesis of cholesterol by activating the AMPK signaling pathway, the thyroid hormone signaling pathway, and the glucagon signaling pathway.

## Graphical abstract

Extracts of *Sanghuangporus vaninii* can relieve hyperlipidemia in rats. The results of transcriptomic analysis indicated that *S. vaninii* extract can activate the AMPK signaling pathway, which subsequently promotes the metabolism of lipids and inhibits the biosynthesis of cholesterol, meanwhile facilitating the excretion of cholesterol, exciting the glucagon signaling pathway and affecting the thyroid hormone signaling pathway, which all contribute to the observed anti‐hyperlipidemic effect.

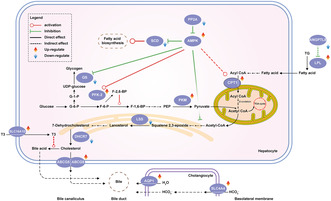

## 1 INTRODUCTION

Hyperlipidemia is a serious risk factor for cardiovascular diseases, including atherosclerosis and coronary artery disease (Nelson, 2013). The clinical manifestation of hyperlipidemia is abnormally increased levels of total cholesterol (TC), triglyceride (TG), and low‐density lipoprotein‐cholesterol (LDL‐C), often accompanied by reduced levels of high‐density lipoprotein‐cholesterol (HDL‐C) (El‐Tantawy & Temraz, 2019). This condition is considered as an important global public health problem of rising incidence combined with a decreasing age of patients (Yang et al., 2020). The WHO estimated that the global prevalence of raised total cholesterol among adults was 39% in 2008 (Pirillo et al., 2021). In 2015–2016, the prevalence of dyslipidemia in American adults over 20 years old was 45.4% (Kosmas et al., 2023). In 2020, the prevalence of hyperlipidemia among Chinese adults over 18 years was 35.6% (Li et al., 2023). According to statistics, the number of global deaths due to hyperlipidemia was 4.3 million in 2019, ranking second in the number of deaths from metabolic diseases (Chew et al., 2023). Several lipid‐lowering drugs have been developed for the clinical treatment of hyperlipidemia; however, side effects of current therapies are often reported, such as hepatotoxicity, nephrotoxicity, and diabetes (Pan et al., 2021). Hence, the development of more effective drugs that can lower lipid blood levels with fewer side effects is wanted.

Edible and medicinal mushrooms that are collectively known as “Sanghuang” are frequently used in Traditional Chinese Medicine (Wan et al., 2020). Modern pharmacological studies showed that Sanghuang can have anti‐oxidation, anti‐inflammatory, and anti‐tumor properties; it has been applied to the treatment of gout and Parkinson's disease, and can lead to hepatoprotection, immunoregulation, and hypoglycemic properties (Guo et al., 2021; Li et al., 2022; Sun et al., 2022; Zhou et al., 2020). It has broad application prospects in the fields of food supplements and natural drugs. The description of Sanghuang constituents in ancient literatures is often unclear, and the term has been used to describe products derived from the species *Phellinus linteus* (homotypic synonyms *Inonotus linteus*, *Tropicoporus linteus*) but more commonly Sanghuang contains species of the genus *Sanghuangporus* including *Sanghuangporus baumii* (homotypic synonyms *Inonotus baumii*, *Phellinus baumii*), *Sanghuangporus sanghuang* (homotypic synonym *Inonotus sanghuang*), or *Sanghuangporus vaninii* (basonym *Phellinus vaninii*, homotypic synonym *Inonotus vaninii*) (Wu & Dai, 2020). At present, there are 14 species in the genus *Sanghuangporus* and of these *S. vaninii* is the main species applied in the health care and medical field because it produces higher yields, although the research on its pharmacological properties is not thorough (Guo et al., 2022; He et al., 2021; Wu & Dai, 2020). There are a few reports that *S. vaninii* has the potential to reduce blood lipids (Huang, Huang, et al., 2022; Huang, Liu, et al., 2022). However, the underlying anti‐hyperlipidemia mechanism of *S. vaninii* is still unclear.

RNA sequencing (RNA‐seq) is applied to reveal the transcriptome profile of organisms or their cells under certain conditions, and this is widely used in the medical field to clarify the pharmacological mechanism of drugs, especially those with complex components and affecting multiple targets, such as Traditional Chinese Medicines (Chen et al., 2022; Wang, Qi, et al., 2021; Wang, Zhuang, et al., 2021). RNA‐seq was shown to be appropriate to demonstrate properties of hypolipidemic agents at the transcriptional level: for example, Hou et al. compared the transcriptome of diet‐induced hyperlipidemic rats and those receiving treatment with mung bean coat, which demonstrated that the PPAR signaling pathway was affected by the treatment (Hou, Liu, et al., 2021). In another example, Tu et al. (2019) investigated the lipid‐lowering efficacy of red raspberry extract and used RNA‐Seq to detect the underlying mechanisms, which identified 7 genes whose expression was closely related to the pharmacological property. Such studies to demonstrate the effect of *S. vaninii* on the transcriptome profile of hyperlipidemic rats has not been reported.

Here, we have used hyperlipidemic rats to validate lipid‐lowering activities of *S. vaninii*. Serum and liver lipid levels of these animals were determined to assess any therapeutic activities, and the transcriptomic analyses were used to explore the influence of *S. vaninii* on hepatic gene expression. Bioinformatic analyses of obtained transcriptomes revealed the probable mechanism of *S. vaninii* responsible for reduced hyperlipidemia.

## 2 MATERIALS AND METHODS

### 2.1 Materials and instruments

*Sanghuangporus vaninii* fruiting bodies were collected from Changbai Mountain and authenticated by Yupeng Cheng. GPS coordinates of the collection place are 42°30′48″N, 129°02′27″E. A voucher specimen (HLJUCM‐CYP‐016) was stored in Biotechnology Laboratory, Heilongjiang University of Chinese Medicine, China.

Simvastatin was purchased from Shanghai Aladdin Biochemical Technology Co., Ltd. Food‐grade lard oil and propylene glycol (purity > 99%) were purchased from Tianjin Ruijinte Chemical Co., Ltd. Food‐grade cholesterol (purity > 99%), sodium cholate (purity > 99%), methylthiouracil (purity > 99%), and yolk powder were purchased from Tianjin Fuyu Fine Chemical Co., Ltd. Standard chow was purchased from Liaoning Changsheng biotechnology Co., Ltd. which contains crude protein, 20%; crude fat, 4%; carbohydrate, 68%; and vitamins and minerals, 3%. All the assay kits for biochemical parameters were purchased from Nanjing Jiancheng Bioengineering Institute.

Allegra 64R centrifuge was purchased from Beckman Coulter, Inc. LyoQuest laboratory freeze dryers were purchased from Azbil Telstar Technologies, S.L.U. RT‐6000 microplate reader was purchased from Rayto Life and Analytical Sciences Co., Ltd. BX‐43 microscope was purchased from Olympus Corporation. RM2235 manual rotary microtome was purchased from Leica Biosystems. LineGene 9620 quantitative PCR system was purchased from Hangzhou Bioer Technology Co., Ltd.

### 2.2 Preparation of *S. vaninii* extracts

*Sanghuangporus vaninii* fruiting bodies (1000 g) were added to distilled water (1:10, w/v), incubated for 0.5 h and then heated to reflux for 1 h, after which it was filtered to obtain the extracted solution. The residue was added to distilled water, refluxed, and filtered again, as described above. Both extracted solutions were combined and freeze dried with a vacuum freeze dryer. A total of 213.5 g freeze‐dried SVE was obtained, and each gram SVE was equivalent 4.68 g fresh mushroom. Then the products were redissolved in distilled water to give a final concentration of *Sanghuangporus vaninii* extract (SVE) of 800 mg mL^−1^ with reference to the amount of fruiting bodies used. This SVE was used for intragastric administration.

### 2.3 Animals and experimental designs

All animal experiments complied with international guidelines (NIH Publication, revised 2011) and were approved by the Institutional Animal Care and Use Committee of Heilongjiang University of Chinese Medicine (Permission No.: HLJUCM201808017). Forty‐eight male Sprague–Dawley rats (weight 200 ± 20 g) (Experimental Animal Center of Heilongjiang University of Chinese Medicine, Haerbin, China) were maintained at 23 ± 2°C, 50 ± 10% relative humidity, with a 12 h photoperiod, with water and fed ad libitum with standard chow. After 1 week of acclimatization, 8 animals were randomly selected as the normal control group (NC) to continue with this standard feeding regime. The other 40 rats moved to a high‐fat diet (HFD) containing 66.5% standard chow, 20% lard oil, 10% yolk powder, 2% cholesterol, 1% sodium cholate, and 0.5% methylthiouracil (The sodium cholate and methylthiouracil were added for increasing the absorption of cholesterol and accelerating the formation of hyperlipidemia.) (Du et al., 2021; Shi et al., 2020). At the end of the 4th week, tail vein blood samples were collected for serum TC, TG, LDL‐C, and HDL‐C level testing to ensure the induction of hyperlipidemia had been successful. These hyperlipidemic rats were randomized into five groups of eight animals that were treated as follows: the hyperlipidemia group (Model) was continuously fed with HFD only and the positive control group (PC) was fed with HFD supplemented with 8 mg kg^−1^ day^−1^ simvastatin. The other three groups received HFD supplemented with low‐dose SVE (L‐SVE: 200 mg kg^−1^ day^−1^ SVE), middle‐dose SVE (M‐SVE: 400 mg kg^−1^ day^−1^ SVE), or high‐dose SVE (H‐SVE: 800 mg kg^−1^ day^−1^ SVE). All doses were administered daily by gavage in 1 mL/100 g bodyweight. During the treatment, food intake was recorded daily. At the end of the 8th week, fasting for 12 h was followed by anesthesia with 3% (w/v) sodium pentobarbital (50 mg kg^−1^ body weight, i.p.). Abdominal aortic blood samples were collected, kept at room temperature for 1 h, and centrifuged (3500 rpm, 4°C, 10 min) to obtain serum. Livers were rapidly removed and weighed. One part of each liver was fixed with 4% paraformaldehyde for histological investigation, and two other two parts were snap‐frozen in liquid nitrogen and maintained at −80°C for biochemical and transcriptome analyses. A liver index was calculated as follows: Liver index = weight of liver/weight of body × 100% (Yadikar et al., 2022).

### 2.4 Biochemical assay of serum and liver

The TC, TG, LDL‐C, and HDL‐C levels of serum and liver tissue were quantified using commercial ELISA kits (Nanjing Jiancheng Bioengineering Institute, China).

### 2.5 Histological examination

Fixed liver samples were dehydrated in an ethanol series and paraffin‐embedded prior to sectioning (5 μm) and staining with hematoxylin–eosin (H&E). Frozen liver samples were cut into 8–10‐μm section and stained with Oil red O solution. The histological sections were examined by light microscopy. The relative area of fat vacuoles and the relative area of lipid droplets were calculated by Image J (Mehlem et al., 2013).

### 2.6 Transcriptome analysis

0.1 g of liver tissue was taken from each rat, then the tissues from the same group of rats were pooled together as samples. Total RNA from liver samples was isolated using TRIzol reagent (Invitrogen, USA) and mRNA purified using poly‐T magnetic beads. After fragmentation, mRNA was reverse transcribed to create cDNA, which was the template for second‐stranded DNA synthesis. DNA was ligated with adapters and amplified to obtain a final cDNA library for sequencing, with an average insert size of 300 ± 50 bp. RNA sequencing was performed on an Illumina Novaseq™ 6000 platform. Hisat software was used to compare reads with the rat reference genome. StringTie software was applied to align transcripts and calculate the number of fragments per kb of transcript per million mapped reads (FPKM). Differentially expressed genes (DEGs) were called using a |log_2_ fold‐change| ≥ 1 and *p* < .05 by means of the edgeR package (Robinson et al., 2010). DEG function (biological function, metabolic pathways, signaling pathways) was analyzed using Gene Ontology (GO) and Kyoto Encyclopedia of Genes and Genomes (KEGG) with ClueGO software (Bindea et al., 2009), Protein–protein interaction networks of putative proteins encoded by DEGs were constructed with STRING (https://cn.string‐db.org/).

### 2.7 Real‐time quantitative PCR validation

For real‐time quantitative evaluation of gene expression, RNA was extracted from liver samples with the SV Total RNA Isolation System (Promega, USA), and reverse transcribed using the GoScript™ kit (Promega). Subsequently, the products were amplified using GoTaq® qPCR (Promega) in a quantitative PCR (qPCR) system (LineGene 9620, Hangzhou Bioer Technology Co., Ltd, China). The primer sequences of the genes of interest are summarized in Table 1. Gene expression was calculated based on the $2^{-∆∆C_{t}}$ method, with *Actb* as internal reference gene (Livak & Schmittgen, 2001).

**TABLE 1:** The primer sequences used for qPCR.

Gene	Forward primer (5′–3′)	Reverse primer (5′–3′)	Length (bp)
Lss	TTGCTACCTATGAGACTAAGCG	GGAGGTACACTCTACATACGTG	107
Dhcr7	CAGATTTCTGCCAGGTTATGTG	CAGCCCATTCACCTCATACTTA	88
Abcg5	CGCAGGAACCGCATTGTAA	TGTCGAAGTGGTGGAAGAGCT	67
Abcg8	CTTGTGAAACAGGTACTTCGTG	CTGCATAGCCTAGCTACTCAAT	120
Aqp1	TGAGATCATTGGCACCCTG	CCAGTGTAGTCAATGGCCA	141
Slc4a4	TTCATTGCCTTTGTTCGTCTAC	GTCGTGTCTATCTTTCGCTTTG	201
Lpl	GATGATGCGGATTTCGTAGATG	TATCAACATGCCCTACTGGTTT	94
Angptl8	GAGGTGGTTCTGCTTATCGG	GTGTACGGAGACTACAAGTGC	101
Prkab2	GTCTTCATCTCTGGGTCTTTCA	CCACAAAGAACTTGTACTGGTG	121
Cpt1b	GTTCACGCCATGATCATGTATC	GAACATCCTCTCCATCTGGTAG	108
Ppp2r3a	GACATCTTTGCGAAGGGACC	GACTTCAGCCTCTTCTTAAACCG	102
Scd	GAGGCAGCATCTGTAAGTAGAT	GAAGCCACGTTTCTACTCAATC	138
Pkm	AAGGAGATGATTAAGTCTGGGA	GATAGTCTCTGCATGGTACTC	81
Pfkfb2	GGTATCCAGAGGAATTTGCAC	CCAGGTCCTGGTATGACTC	81
Gys1	GCCCATGTCTTCACTACAG	GGTCACAATATCTGGTTTCCT	81
Slc16a10	AACTTTGCCCTCTTCAAGG	CCTTTACATGGTTCATCAAGTG	109
Actb	ACGGTCAGGTCATCACTATCG	GGCATAGAGGTCTTTACGGATG	155

### 2.8 Statistical analysis

Data are given as mean ± SD. Student's *t*‐test and one‐way analysis of variance (ANOVA) were applied to compare biochemical parameters and qPCR results between different groups. Fisher's exact test was used for bioinformatic analysis, with *p* < .05 considered statistically significant.

## 3 RESULTS

### 3.1 Confirmation of the applied hyperlipidemia rat model

**FIGURE 1:**
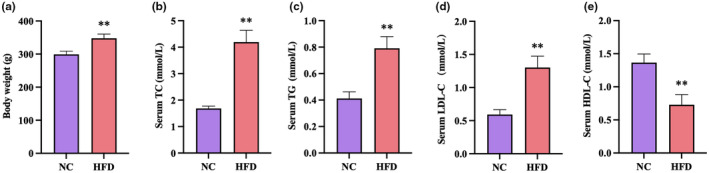
Body weight and serum lipid parameters of the normal control group (NC) and of rats fed a high‐fat diet (HFD) for 4 weeks. (a) body weight, (b) serum TC, (c) serum TG, (d) serum LDL‐C, (e) serum HDL‐C. Data are displayed as mean ± SD, *n* = 8 for NC, *n* = 40 for HFD. **p < .01 versus NC group.

After 4 weeks of induction of hyperlipidemia with the high‐fat diet, the body weight of the rats was significantly higher (*p* < .01) than in the NC group receiving normal chaw (Figure 1). The increased levels of serum TC, TG, and LDL‐C (*p* < .01), together with reduced HDL‐C (*p* < .01) in the rats on the high‐fat diet confirmed induction of hyperlipidemia in these animals.

### 3.2 Effects of SVE on food intake, body weight, and liver index

**FIGURE 2:**
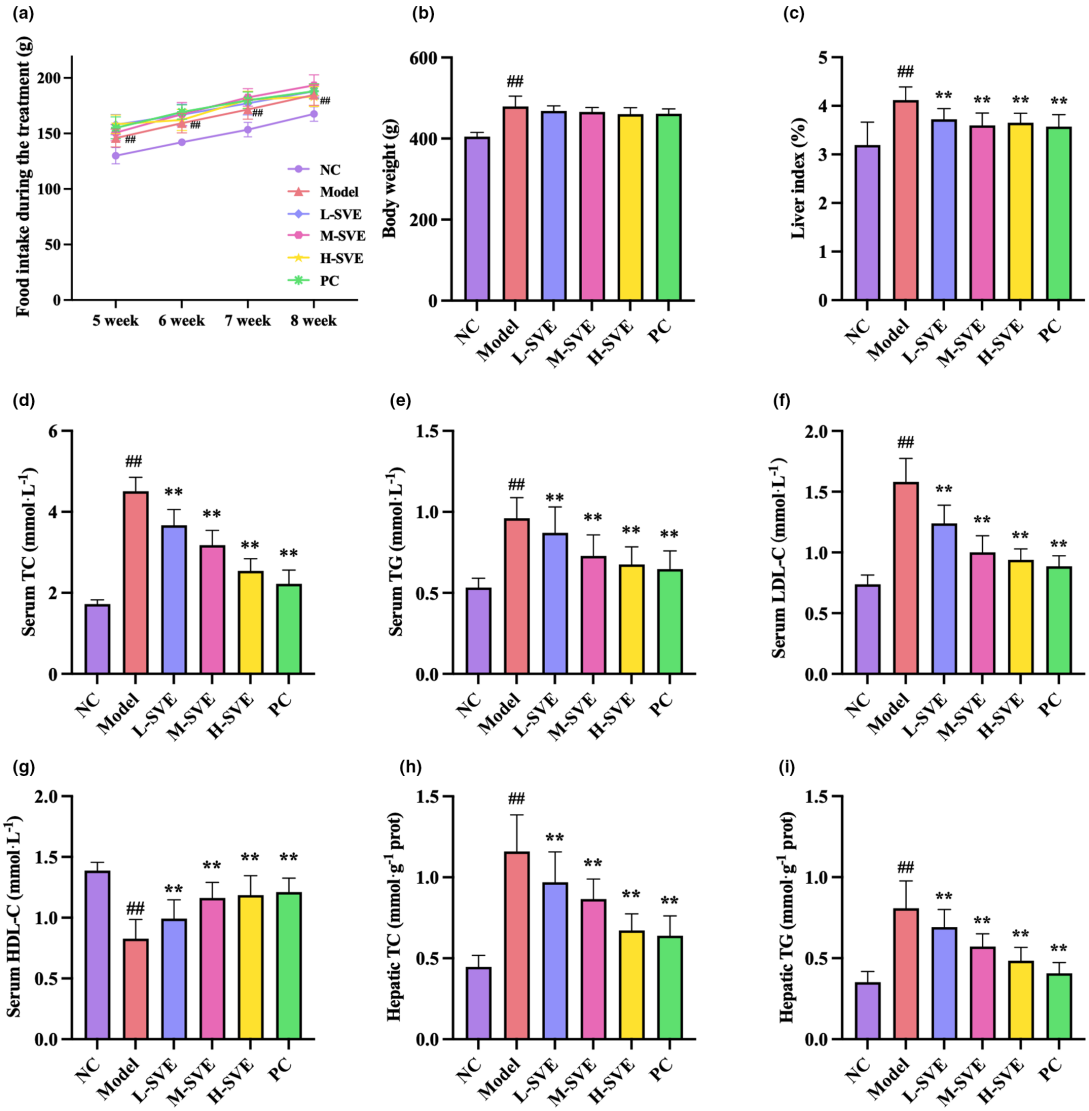
Effects of SVE treatment combined with a HFD diet on food intake, body weight, liver index, and lipid parameters in hyperlipidemic rats. (a) Food intake, (b) Body weight, (c) Liver index, (d) Serum TC, (e) Serum TG, (f) Serum LDL‐C, (g) Serum HDL‐C, (h) Hepatic TC, (i) Hepatic TG. Data are displayed as mean ± SD, *n* = 8 for all groups. ^##^ *p* < .01 versus NC group, **p < .01 versus Model group.

The hyperlipidemic rats were treated for 4 weeks with three doses of SVE under continuous HFD feeding, with inclusion of proper controls (Model, PC). The food intake of rats in all groups increased with time. The food intake of the Model group rats was significantly higher than that of the NC group (*p* < .01), while there was no significant difference in food intake between the treatment groups and the Model group rats (Figure 2a). After 4 weeks of administration, body weight in the Model, PC, L‐SVE, M‐SVE, and H‐SVE groups was the same (Figure 2b). The liver index in the Model group increased, while treatment with SVE and simvastatin had obviously reduced the liver index; however, there were no differences in liver index between the treatment groups (Figure 2c).

### 3.3 Effects of SVE on the serum lipid profiles and hepatic lipid accumulation

After the 4‐week treatment, the serum levels of TC, TG, and LDL‐C were higher in Model than in NC mice (*p* < .01), while HDL‐C was lower (*p* < .01) in the Model group (Figure 2d–i). The serum TC, TG, and LDL‐C levels in the PC and SVE treatment groups were all lower (*p* < .01) than in the Model group, and HDL‐C levels in PC and SVE treatment groups were higher (*p* < .01). The lipid‐lowering activity of SVE treatment exhibited a dose‐dependent relationship, whereby the serum lipid levels in H‐SVE and PC groups were similar. In liver tissues, levels of TC and TG were decreased in SVE treatment compared with the Model groups (*p* < .01), depending on dose.

### 3.4 Effects of SVE on liver histopathology

**FIGURE 3:**
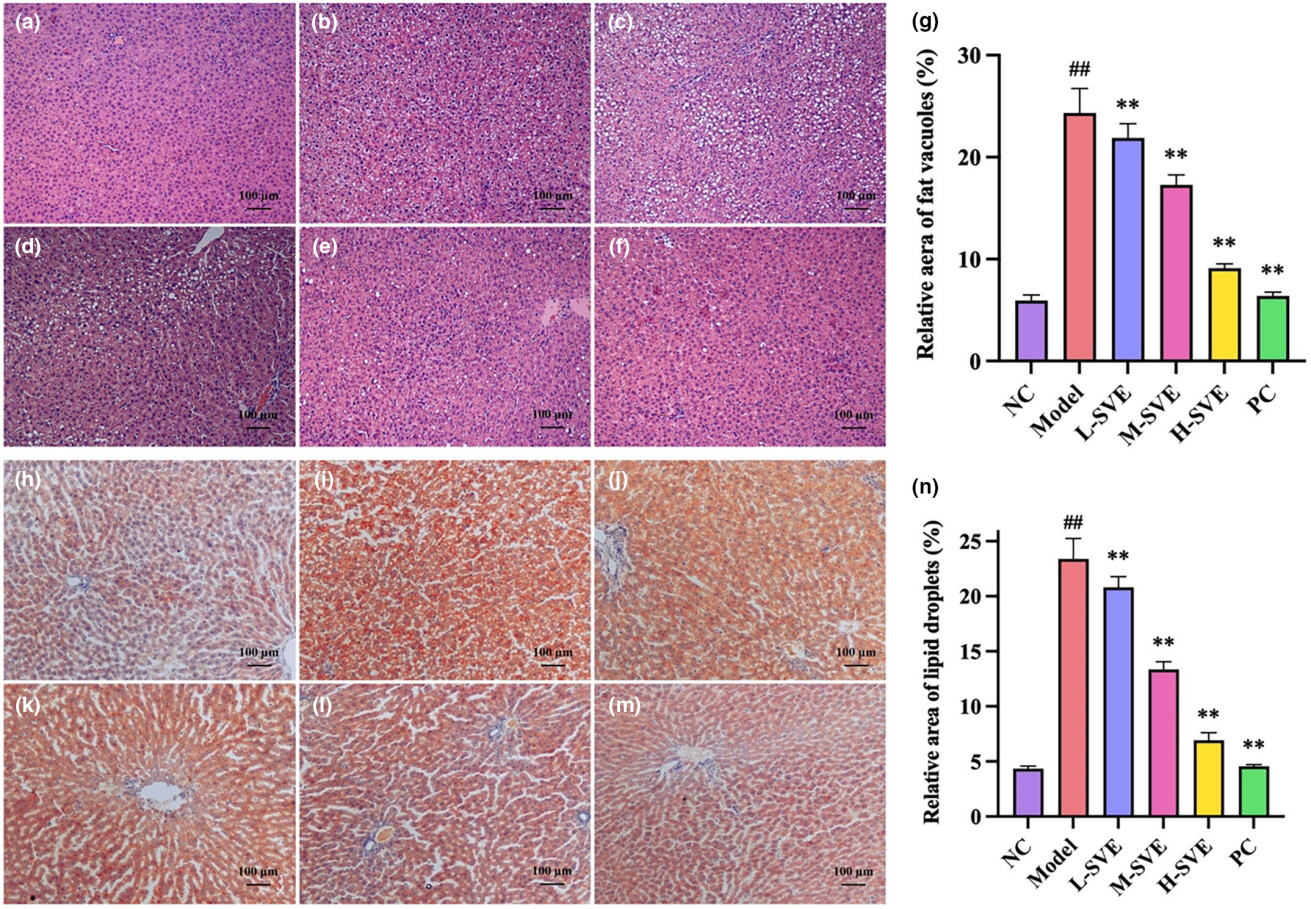
Effects of SVE on histopathological changes in livers by H&E staining and Oil red O staining (magnification, 100×; scar bar represent 100 μm). (a) NC group by H&E staining, (b) Model group by H&E staining, (c) L‐SVE group by H&E staining, (d) M‐SVE group by H&E staining, (e) H‐SVE group by H&E staining, (f) PC group by H&E staining, (g) relative area of fat vacuoles, (h) NC group by Oil red O staining, (i) Model group by Oil red O staining, (j) L‐SVE group by Oil red O staining, (k) M‐SVE group by Oil red O staining, (l) H‐SVE group by Oil red O staining, (m) PC group by Oil red O staining, (n) relative area of lipid droplets. Data are displayed as mean ± SD, *n* = 8 for all groups. ^##^ *p* < .01 versus NC group, **p < .01 versus Model group.

Liver tissue sections were stained with H&E to detect any histopathological differences among the groups. In the NC group, the hepatic structure was arranged orderly and no abnormalities were observed. In contrast, there was obvious structural damage of hepatocytes and accumulation of lipid droplets in Model group livers, while the SVE and PC group livers exhibited fewer fat vacuoles and produced milder hepatic steatosis in their liver tissue (Figure 3a–g). The Oil red O staining also showed that after treatment with SVE, the content of oil droplets was significantly decreased compared with the model group (Figure 3h–n). The staining results indicated that treatment with SVE alleviated the hepatic steatosis in a dose‐dependent way.

### 3.5 Effects of SVE on transcriptome profile

**FIGURE 4:**
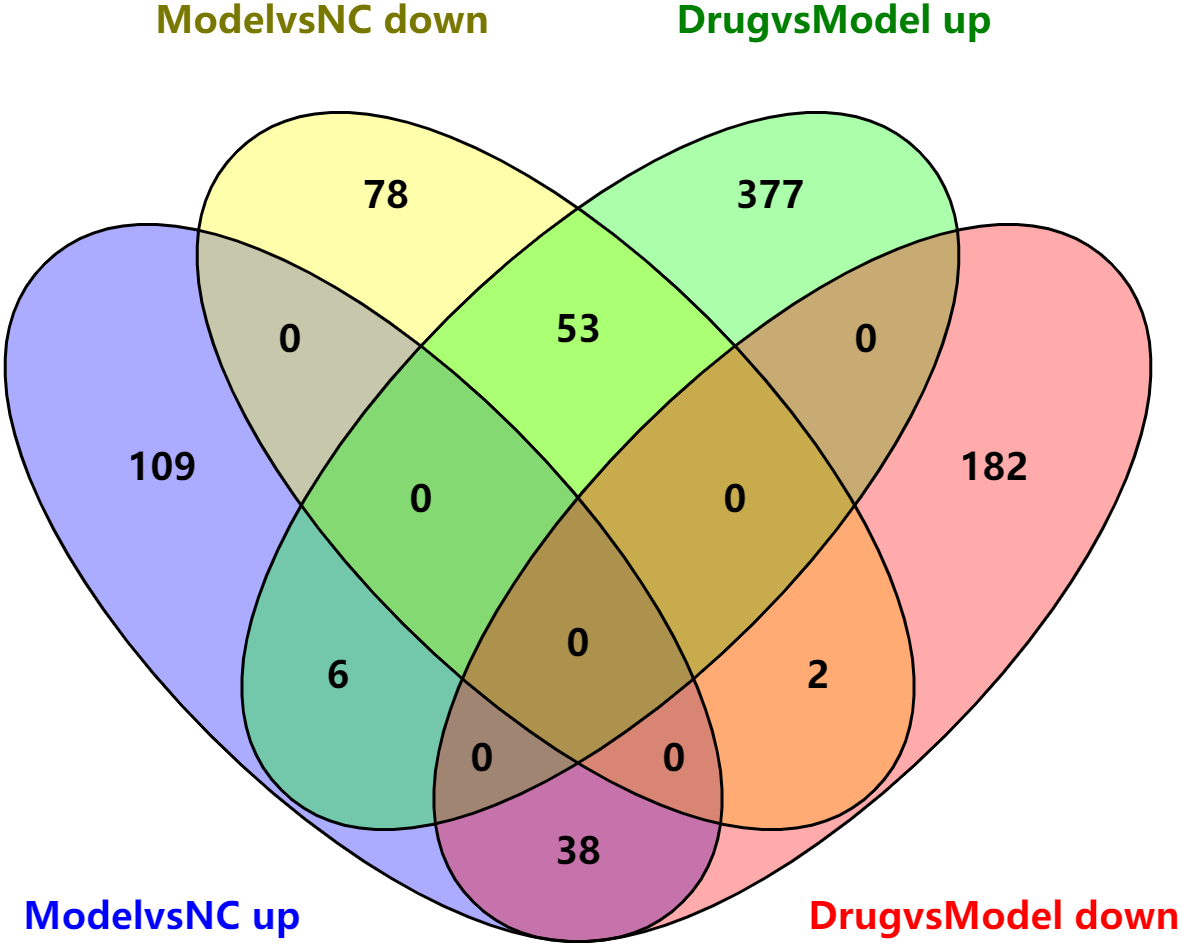
Differentially expressed genes identified among the NC, Model, and H‐SVE treatment groups. “ModelvsNC up” shows genes up‐regulated in the Model group relative to NC; “ModelvsNC down” shows genes down‐regulated in the Model group relative to NC. Likewise, “DrugvsModel up” and “DrugvsModel down” shows genes up‐ or down‐regulated, respectively, in the H‐SVE group relative to the Model group.

To reveal potential mechanisms underlying SVE's lipid‐lowering activity, transcriptome profiles were analyzed by RNA‐Seq. This identified differently expressed genes between the Model and NC groups, as well as between the H‐SVE and Model groups. Using the NC group for comparison, 286 DEGs were identified in the Model group, including 153 up‐regulated and 133 down‐regulated genes; compared with the Model group, 658 DEGs were identified in the H‐SVE group, with 436 up‐regulated and 222 down‐regulated genes. A combined analysis showed that there were 91 common genes in total, of which 38 were up‐regulated in the Model group while down‐regulated in the H‐SVE group, and the remaining 53 exhibited the opposite changes in expression (Figure 4, Table S1).

### 3.6 Functional enrichment and network analysis of DEGs in the model versus NC groups

**FIGURE 5:**
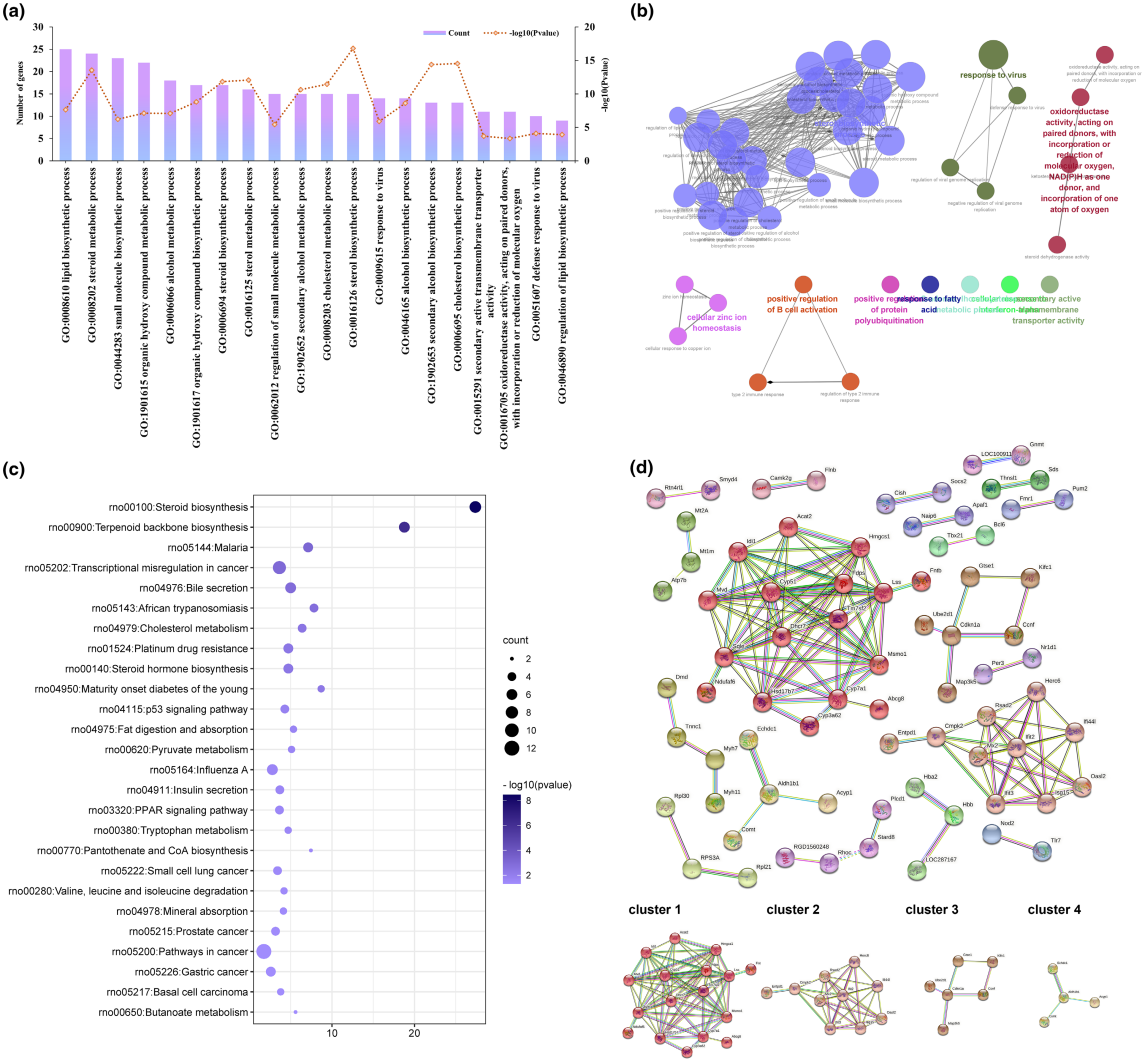
Transcriptomic analysis of DEGs in the Model versus NC group. (a) Bar chart of the top 20 biological process terms based on GO enrichment results. (b) Network diagram showing correlations of the enriched biological process terms. (c) Bubble plot showing KEGG enrichment results. (d) The protein–protein interaction network of DEGs in the Model versus NC group with the 4 largest clusters based on the Markov clustering (MCL) shown at the bottom.

GO analysis of identified DEGs in the Model and NC groups was performed to explore the variation of their biological functions. The three main categories in the GO system—biological processes (BP), cellular components (CC), and molecular functions (MF)—describe the biological domains with respect to the larger processes accomplished by multiple molecular activities, the positions within cellular structures where the gene products function, and the activities carried out by the gene products at the molecular level, respectively. For DEGs belonging to the category of BP, those of the Model group versus the NC group were implicated in 49 GO terms which were clustered into 10 groups (Table S2). The top 20 BP terms covered by the most genes are exhibited in Figure 5a, and, with high relevance to hypolipidemia, the major groups involved in lipid metabolism were sterol biosynthetic process and response to fatty acid, as shown in the relationship network of GO terms (Figure 5b). Pathways involved in DEGs that were enriched based on KEGG were also identified, with a total of 26 metabolism or signaling pathways (Table S3). Of these, the pathways associated with lipid metabolism were steroid biosynthesis (rno00100), terpenoid backbone biosynthesis (rno00900), bile secretion (rno04976), cholesterol metabolism (rno04979), fat digestion and absorption (rno04975), and PPAR signaling pathway (rno03320) (Figure 5c). A putative protein–protein interaction (PPI) network between DEGs was determined using STRING, setting the interaction score parameter to high confidence in order to elucidate the correlation among them. The network returned 74 nodes and 134 edges. Twenty clusters were obtained based on the Markov clustering (MCL) (Table S4), and the largest cluster contained 17 genes whose functions mainly referred to the regulation of lipid metabolisms, such as steroid biosynthesis (rno00100), fat digestion and absorption (rno004975), and cholesterol metabolism (rno04979) (Figure 5d, Table S5).

### 3.7 Functional enrichment and network analysis of DEGs in the H‐SVE versus model group

**FIGURE 6:**
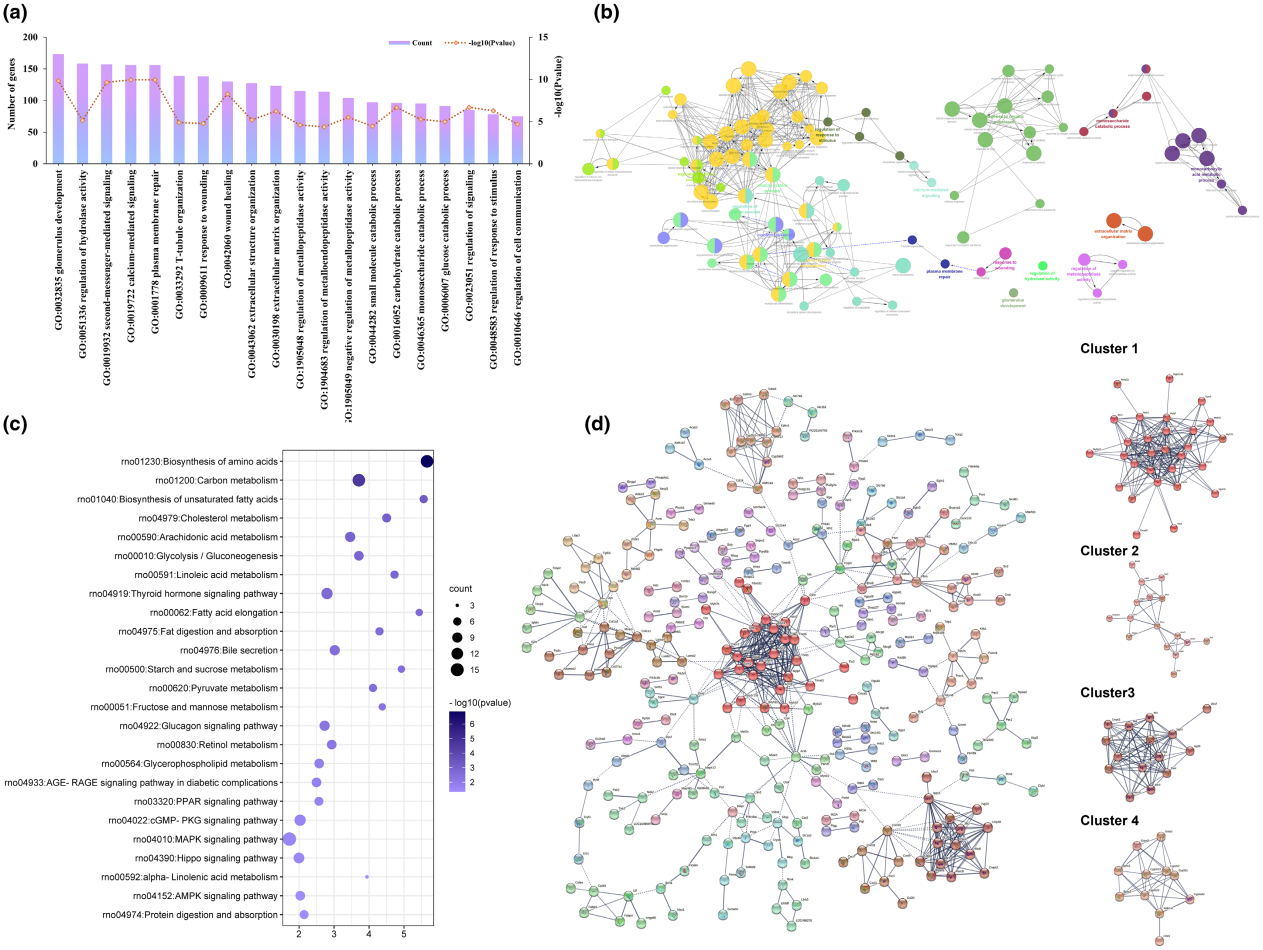
Transcriptomic analysis results of DEGs in the H‐SVE versus Model group. (a) Bar chart of the top 20 biological process terms based on GO enrichment. (b) Correlations of the enriched biological process terms. (c) The KEGG enrichment. (d) The PPI interaction network of the DEGs with the 4 largest clusters based on the MCL shown at the right.

The result of GO enrichment of DEGs in the H‐SVE versus the Model group showed that 101 terms belonging in the category of BP were enriched, and these terms were further clustered into 16 groups (Table S6). The top 20 biological process terms are displayed in Figure 6a, with processes involved in lipid metabolism identified as monocarboxylic acid, regulation of response to stimulus, and regulation of hydrolase activity (Figure 6b). KEGG pathway analysis revealed that DEGs were enriched in 57 metabolic or signaling pathways (Table S7). The main pathways associated with lipid metabolism were biosynthesis of unsaturated fatty acids (rno01040), cholesterol metabolism (rno04979), fatty acid elongation (rno00062), fat digestion and absorption (rno04975), bile secretion (rno04976), PPAR signaling pathway (rno03320), and AMPK signaling pathway (rno04152) (Figure 6c). The PPI network of these DEGs contained 293 nodes and 613 edges, and all nodes were clustered into 75 clusters (Table S8). As shown in Figure 6d, cluster 2 contained 16 genes whose functions were mainly related to biosynthesis of unsaturated fatty acids (rno01040), fatty acid elongation (rno00062), AMPK signaling pathway (rno04152), and PPAR signaling pathway (rno03320), whereas cluster 4 contained 11 genes whose functions were mainly related to steroid hormone biosynthesis (rno00140), arachidonic acid metabolism (rno00590), and metabolism of xenobiotics by cytochrome P450 (rno00980) (Tables S9 and S10).

### 3.8 Identification of potential pathways and genes involved in the antihyperlipidemic activity of H‐SVE

**FIGURE 7:**
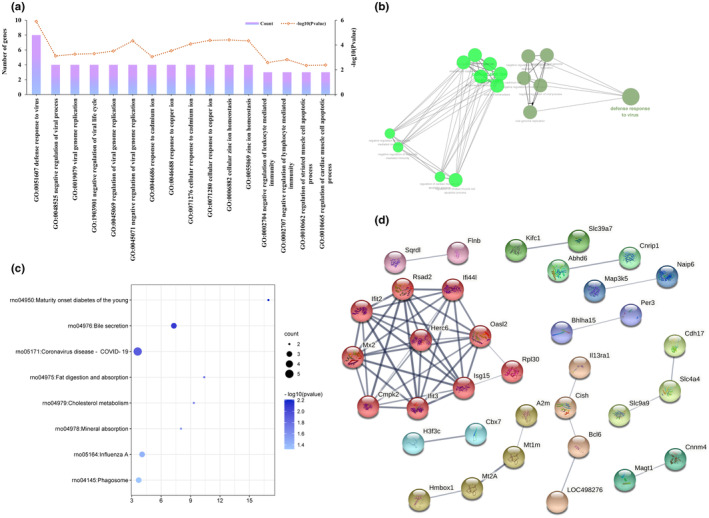
Transcriptomic analysis results of 91 common genes. (a) The biological process enrichment (b) Correlation network of the enriched biological process terms. (c) The KEGG enrichment results. (d) PPI network.

**FIGURE 8:**
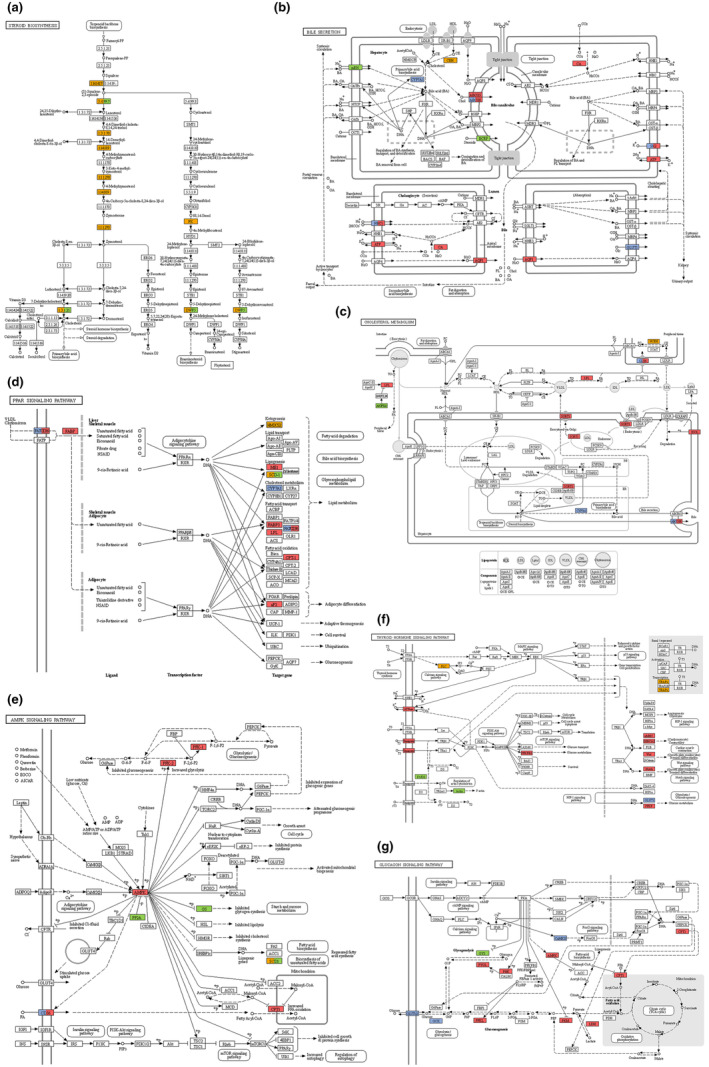
The pathways participating in the lipid‐lowering activity of H‐SVE treatment. (a) Steroid biosynthesis, (b) Bile secretion, (c) Cholesterol metabolism, (d) PPAR signaling pathway, (e) AMPK signaling pathway, (f) Thyroid hormone signaling pathway, (g) Glucagon signaling pathway. Different colors represent different trends in gene expression, with orange and blue for up‐ and down‐regulated gene expression, respectively, in the Model group compared with the NC; red and green represent up‐ and down‐regulated gene expression, respectively, in the H‐SVE group compared with the Model group; blank means no significant difference in expression was detected.

GO enrichment, KEGG enrichment, and PPI network construction were also applied to analyze the identified 91 common genes. These genes were enriched in 16 GO biological process terms and 8 KEGG pathways (Table S11, Figure 7a–c). The PPI network is depicted in Figure 7d. A combination of all three analyses of the DEGs (Model vs. NC group, H‐SVE vs. Model group, and common genes) showed that long‐term feeding on a HFD can disrupt lipid metabolism in the rat by promoting steroid and fat acid biosynthesis, decreasing bile secretion and inhibiting PPAR signaling pathway, whereas H‐SVE can reversely regulate certain pathways by targeting multiple genes (Figure 8a–d). Meanwhile, H‐SVE also can promote lipid metabolism by stimulating AMPK, thyroid hormone, and glucagon signaling pathways (Figure 8e–g). Comprehensively considering the pathways and PPI networks, it is suggested that the genes *Lss*, *Dhcr7*, *Abcg5*, *Abcg8*, *Aqp1*, *Slc4a4*, *Lpl*, *Angptl8*, *Prkab2*, *Cpt1b*, *Ppp2r3a*, *Scd*, *Pkm*, *Pfkfb2*, *Gys1*, and *Slc16a10* may play key roles in the antihyperlipidemic activity of H‐SVE.

### 3.9 Confirmation of transcriptomic sequencing with qPCR

**FIGURE 9:**
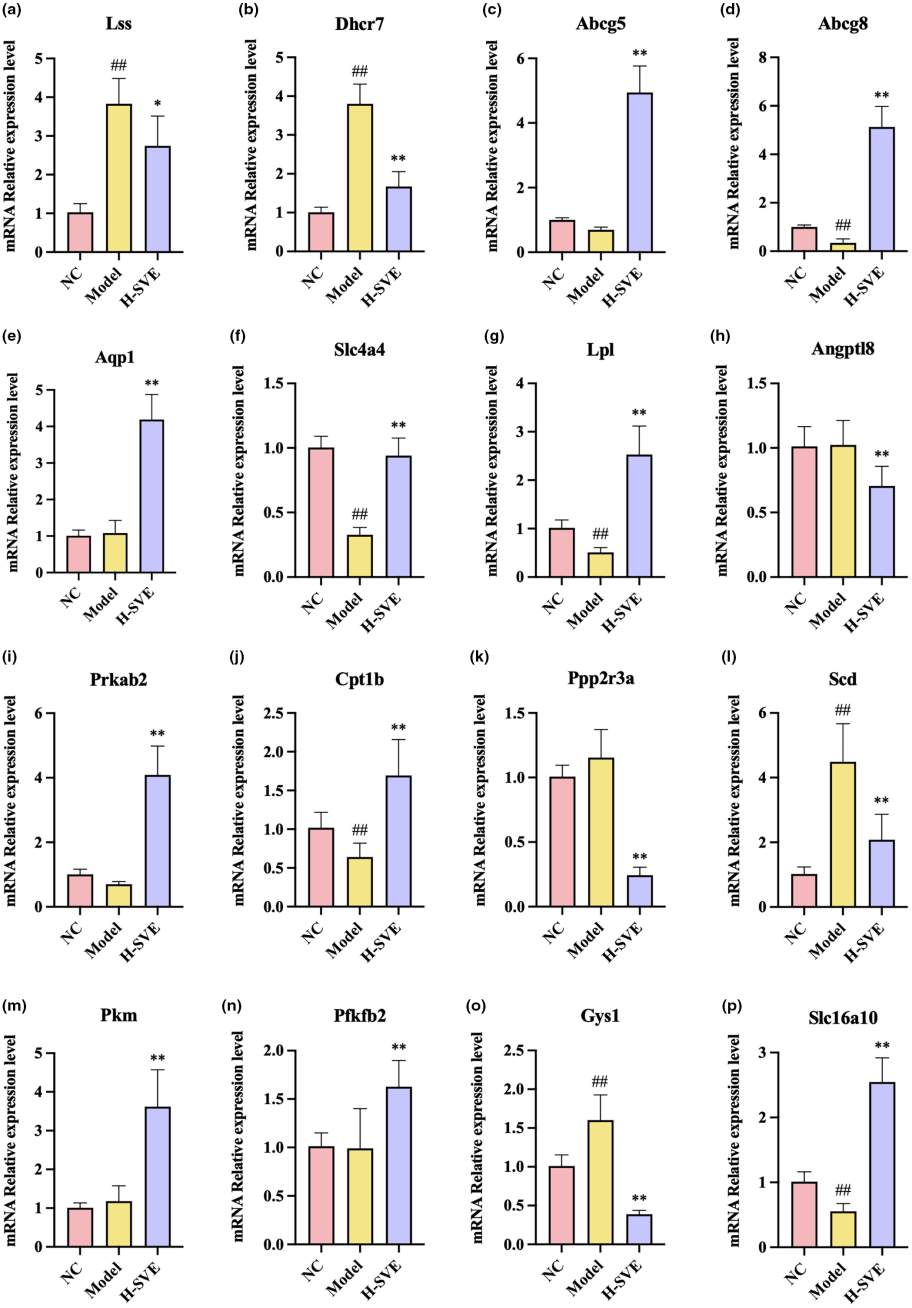
Relative expression levels of candidate genes validated by qPCR. (a) *Lss*, (b) *Dhcr7*, (c) *Abcg5*, (d) *Abcg8*, (e) *Aqp1*, (f) *Slc4a4*, (g) *Lpl*, (h) *Angptl8*, (i) *Prkab2*, (j) *Cpt1b*, (k) *Ppp2r3a*, (l) *Scd*, (m) *Pfkm*, (n) *Pfkfb2*, (o) *Gys1*, (p) *Slc16a10*. Data are displayed as mean ± SD, *n* = 8 for all groups. ^#^ *p* < .05 versus NC group, ^##^ *p* < .01 versus NC group, *p < .05 versus Model group, **p < .01 versus Model group.

To verify transcriptomic results and the effect of SVE treatment on certain genes involved in hyperlipidemia, the relative expression levels of the genes *Lss*, *Dhcr7*, *Abcg5*, *Abcg8*, *Aqp1*, *Slc4a4*, *Lpl*, *Angptl8*, *Prkab2*, *Cpt1b*, *Ppp2r3a*, *Scd*, *Pkm*, *Pfkfb2*, *Gys1*, and *Slc16a10* were determined by qPCR (Figure 9). Compared with the NC group, the expression of *Lss*, *Dhcr7*, *Scd*, *Gys1* was up‐regulated and that of *Abcg8*, *Slc4a4*, *Lpl*, *Cpt1b*, *Slc16a10* was down‐regulated in the Model group. However, these altered gene expression levels were mitigated by H‐SVE treatment. Meanwhile, the expression of *Abcg5*, *Prkab2*, *Aqp1*, *Pkm*, and *Pfkfb2* was up‐regulated, while *Angptl8* and *Ppp2r3a* was down‐regulated in H‐SVE relative to the Model group, with no significant difference between Model and NC groups. This was consistent with the transcriptomic sequencing results and corroborated the RNA‐seq data.

## 4 DISCUSSION

Hyperlipidemia is a condition of abnormally elevated blood lipid levels induced by genetic and external causes. The condition provides a risk for multiple illnesses that may ultimately be lethal (Jia et al., 2021). For this reason, pharmaceutical research has concentrated to identify effective lipid‐lowering drugs which preferably have few side effects. *S. vaninii* is an edible and medicinal mushroom with various efficacy (Guo et al., 2021); its safety and its potential to reduce blood lipid levels make it a likely anti‐hyperlipidemia agent (Huang, Liu, et al., 2022; Huo et al., 2020). In this study, a high‐fat diet was used to induce hyperlipidemia in rats, who were then treated with SVE to confirm the effects of *S. vaninii*, with simvastatin as a positive control. Our results showed that the SVE treatment significantly reduced TC, TG, and LDL‐C serum levels, increased HDL‐C levels, and alleviated the hepatic pathological changes in hyperlipidemic rats to a satisfactory level. Transcriptome analysis revealed the underlying molecular mechanism of this lipid‐lowering activity, which identified that SVE could affect the expression of multiple hepatic genes and pathways related to lipid metabolism.

Among the genes related to lipid metabolism, SVE had a significant effect on the expression of genes involved in cholesterol synthesis and export. Cholesterol, an essential fat, is a component of cell membranes and acts as an important precursor in the synthesis of steroid hormones (Schade et al., 2020). Abnormal cholesterol metabolism is one of the main symptoms of hyperlipidemia. In mammals, cholesterol is synthesized in the liver from acetate, whereby three molecules of acetyl‐CoA combined into mevalonate, which is then transformed by acetyl‐CoA acetyl transferase, HMG‐CoA synthase, and HMG‐CoA reductase in sequence. The formed mevalonate converts to two activated isoprenes, and six of these activated isoprene units condense to form squalene. Squalene is catalyzed by squalene epoxidase (SQLE) to produce squalene 2,3‐epoxide, which is subsequently converted to lanosterol by lanosterol synthase (LSS) (Xu et al., 2020). After multistep reactions, lanosterol is eventually changed to cholesterol, with 7‐dehydrocholesterol reductase (DHCR7) catalyzing the final step (Zou et al., 2022). Current research indicated that LSS was considered as a promising target for lipid‐lowering drugs to avoid the side effects of statins (Batista et al., 2022; Cohain et al., 2021; Trapani et al., 2011), and the decreased expression of *Dhcr7* has also been proved to be beneficial for improving the glycolipid metabolism disorder and lowering cellular cholesterol (Kuwabara et al., 2022; Sun et al., 2023). Taking together the results of transcriptome sequencing and qPCR presented here, it is concluded that the expression of *Lss* and *Dhcr7* was down‐regulated in treatment groups, which may explain how SVE can inhibit the synthesis of cholesterol and reduce its accumulation (Figure 8a).

Most cholesterol is exported from the liver in one of three forms: bile acids, biliary cholesterol, or cholesteryl esters (Xu et al., 2020). Cholesterol is transported between tissues in the form of lipoprotein particles, which is classified into chylomicrons, VLDL, LDL and HDL based on their different densities (Feingold, 2022). Therefore, in addition to inhibiting the synthesis of cholesterol, attention has also been paid to the regulation of biliary secretion and lipoprotein metabolism for treatment of hyperlipidemia. In particular, ATP‐binding cassette sub‐family G members 5 (ABCG5) and 8 (ABCG8) are critical for maintaining sterol homeostasis. These two genes are expressed in the liver and intestine, where they form a heterodimer that mediates the transmembrane transport of cholesterol. The function of ABCG5/ABCG8 in enterocytes is to pump the sterols back into the gum lumen and in hepatocytes to promote the secretion of cholesterol into bile (Zein et al., 2019). It has been shown that overexpression of *Abcg5/Abcg8* increased biliary cholesterol concentrations and reduced cholesterol absorption, which is involved in the efficacy of many lipid‐lowering drugs (Hou, Zhang, et al., 2021; Lee et al., 2021; Yu et al., 2014). Aquaporins (AQPs) belong to a family of membrane channel proteins responsible for the transport of water and other small molecules across the cytoplasmic membrane. AQP1 is the predominant isoform of aquaporins expressed in cholangiocytes and participates in bile production (Banales et al., 2006; Li et al., 2009). In addition, Solute carrier family 4 member 4 (SLC4A4), also known as ${{\mathrm{Na}}^{+}‐\mathrm{HCO}}_{3}^{-}$ cotransportor 1 (NBC1), carbonic anhydrase 2 (CA2), and sodium/potassium‐transporting ATPase subunit alpha‐1 (ATP1A1) are all necessary for intracellular accumulation of ${\mathrm{HCO}}_{3}^{-}$, which is the basis for bicarbonate secretion at the canaliculi and the bile ducts, and thus generates the so‐called bile‐salt independent flow (Uriarte et al., 2010). Here, we demonstrated that SVE treatment up‐regulated the expression of the genes *Abcg5*, *Abcg8*, *Aqp1*, and *Slc4a4*, thereby promoting the biliary secretion and accelerating cholesterol excretion (Figure 8b).

Our results furthermore showed that Lpl expression was promoted and Angptl8 expression reduced (Figure 8c). In lipid metabolism, lipoprotein lipase (LPL) hydrolyzes triglycerides packed in circulating chylomicrons and very low‐density lipoprotein (VLDL) into free fatty acids, so that consequently the triglycerides are cleared from bloodstream and in this way the lipid levels are regulated (Wu et al., 2021). Mutations in the *LPL* gene can lead to type I hyperlipoproteinemia (Pingitore et al., 2016) and LPL is also considered to be a key target for many lipid‐lowering agents (Moon et al., 2022). Similarly, angiopoietin like protein 8 (ANGPTL8), the latest member of the ANGPTL family, is also regarded as a potential target for anti‐hyperlipidemia due to its ability to inhibit LPL activity by interacting with ANGPTL3, and then regulate plasma TG levels (Li et al., 2021; Sylvers‐Davie & Davies, 2021). Based on this, it is suggested the lipid‐lowering activity of SVE was related to its lipoprotein degradation ability.

The results of transcriptome analysis also showed that some signaling pathways involved in regulating lipid metabolism changed significantly. The signaling pathway AMPK appeared pivotal according to the pathway analysis. AMP‐activated protein kinase (AMPK) is considered a major regulator in carbohydrate and lipid metabolism that acts as an energy sensor (Luo et al., 2010). AMPK can be activated by phosphorylation mediated by some upstream kinases, whereas protein phosphatase 2A (PP2A) is a negative regulator of this signaling pathway by catalytic dephosphorylation of AMPK (Park et al., 2013). Once activated, AMPK inhibits fatty acid and glycogen synthesis, and activates fatty acid oxidation and glycolysis, which restrains hepatic lipid accumulation (Kim et al., 2016). Thus, AMPK is thought to be as a promising target for treatment of lipid metabolism disorders, including hyperlipidemia (Kim et al., 2022; Lew et al., 2020). Carnitine palmitoyltransferase 1 (CPT1) is located at the mitochondrial membrane and is responsible for transporting fatty acids into these organelles, where the long‐chain fatty acids are oxidized by catalytic transfer of their acyl group onto carnitine. This process is a committed step for fatty acid β‐oxidation (Zammit, 2008). CPT1 is highly sensitive to the inhibition by malonyl‐CoA, converted from acetyl‐CoA via acetyl‐CoA carboxylase (ACC) (Brownsey et al., 2006). AMPK can inhibit ACC activity through increasing the phosphorylation level of ACC (Xi et al., 2015). Therefore, the activation of AMPK can promote the expression of CPT1, enhance the oxidation of fatty acids, and reduce the lipid accumulation. Stearoyl‐CoA desaturase 1 (SCD1) is also involved downstream of the AMPK signaling pathway. It catalyzes the biosynthesis of monounsaturated fatty acids and is a key lipogenesis regulator (ALJohani et al., 2017). Activated AMPK suppresses the expression of SREBP‐1c, which regulates the transcription of *SCD1*. Hyperexpression of SCD1 is linked with a variety of metabolic disorders, like hypertriglyceridemia, and hence SCD1 inhibitors are attractive candidates to treat metabolic syndrome (Kamal et al., 2018). Here, we have confirmed that *Prkab2* expression (coding for the non‐catalytic subunit beta‐2 of AMPK (Dasgupta et al., 2012)) and of *Cpt1b* was up‐regulated following treatment with SVE, whereas the expression of *Ppp2r3a* (coding for the regulatory subunit B″ subunit alpha of protein phosphatase 2A; Chen et al., 2019) and of *Scd* were down‐regulated (Figure 8e). Previous studies have also shown that hispidin, a phenolic compound isolated from *Phellinus linteus* (one of the former species names for describing Sanghuang), can activate the AMPK signaling pathway (Lee et al., 2017). The results indicated that SVE activated the AMPK signaling pathway and promoted the metabolism of lipids to attenuate hyperlipidemia. In addition, the results showed that *Pkm* (coding for pyruvate kinase, the rate‐limiting enzyme of glycolysis) and *Pfkfb2* (coding for fructose‐2,6‐bisphosphatase, whose enzymatic product fructose 2,6‐bisphosphate is an activator of glycolysis) were up‐regulated, while *Gys1* (coding for glycogen synthase) was down‐regulated in the SVE group. This means that glycolysis was promoted and the synthesis of glycogen was inhibited in the treated animals, providing assistance for regulating the lipid metabolism by SVE.

**FIGURE 10:**
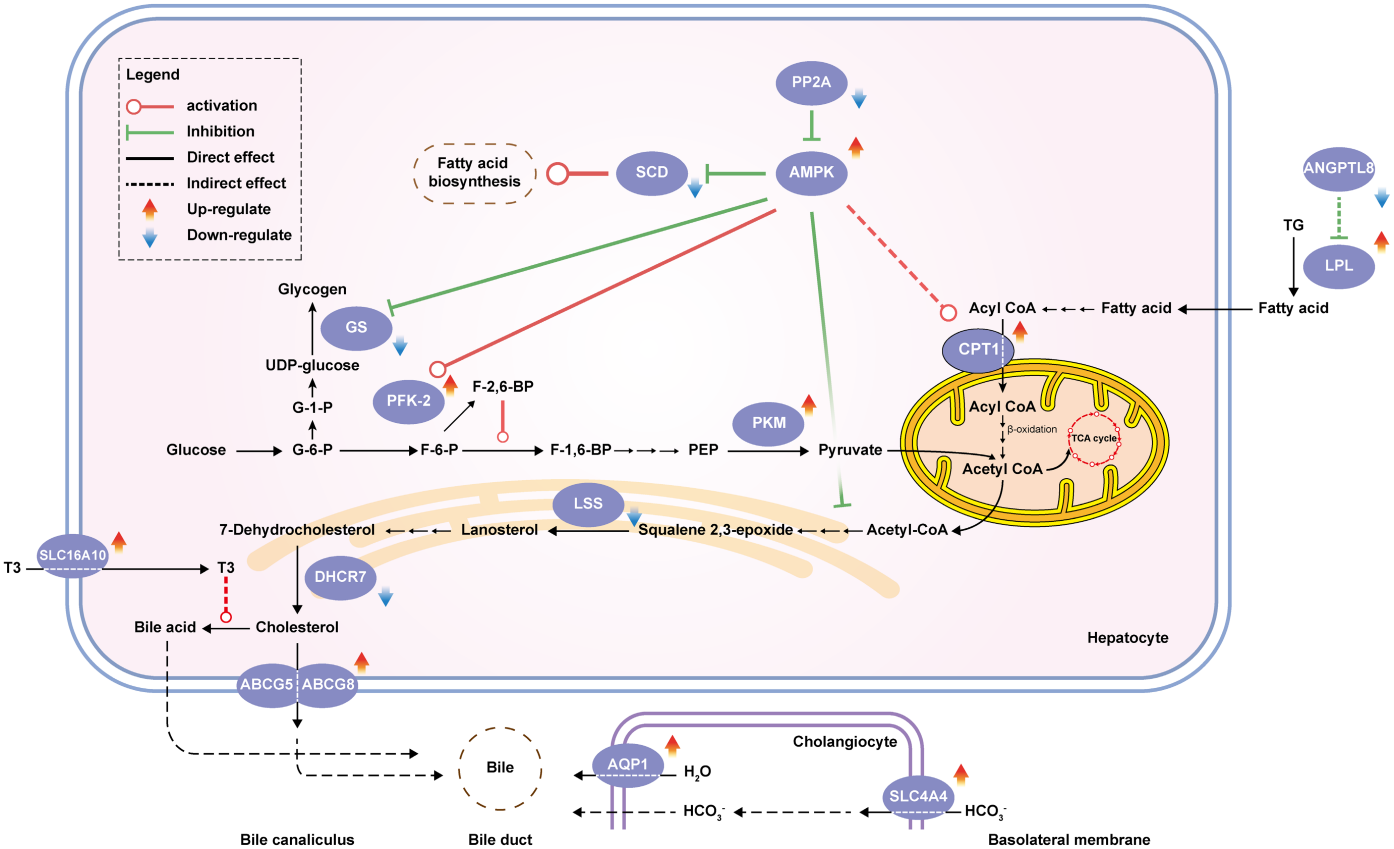
Summary of the potential molecular mechanisms that are involved in the lipid‐lowering activity of *Sanghuangporus vaninii* extract.

In addition, the glucagon signaling pathway (Figure 8f) and thyroid hormone signaling pathway (Figure 8g) were also activated by SVE. The glucagon signaling pathway participates in the lipid metabolism regulation, and its activation can promote lipid degradation and decrease the lipid levels in plasma (Patel et al., 2017; Wang et al., 2016). By analogy, thyroid hormone T3 interacts with intracellular receptors to affect multiple physiological and metabolic processes, including a reduction in the serum levels of LDL‐C, by increasing LDL clearance and LPL activity (Jakobsson et al., 2017; Pucci et al., 2000). In combination, our results indicated that SVE improved lipid profiles by regulating multiple pathways, as summarized in Figure 10.

## 5 CONCLUSION

The dose‐related, lipid‐lowering activity of SVE was confirmed in induced hypolipidemic rats following 4 weeks of treatment. This effect was the result of altered transcription of multiple genes and pathways that improved the lipid profiles of the animals. SVE can activate the AMPK signaling pathway, which subsequently promotes the metabolism of lipids and inhibits the biosynthesis of cholesterol, meanwhile facilitating the excretion of cholesterol, exciting the glucagon signaling pathway, and affecting thyroid hormone signaling pathway, which all contribute to the observed anti‐hyperlipidemic activity. This study revealed the potential mechanism of SVE in treating hyperlipidemia by transcriptomic analysis, and provides strong evidence and support to further explore lipid‐lowering activities of drugs based on extracts of *S. vaninii*.

## AUTHOR CONTRIBUTIONS

**Ning Gao:** Conceptualization (equal); formal analysis (lead); methodology (equal); writing – original draft (lead). **Yuanzhen Liu:** Data curation (equal); investigation (equal). **Guangjie Liu:** Data curation (equal); investigation (equal). **Bo Liu:** Methodology (equal); supervision (lead). **Yupeng Cheng:** Conceptualization (equal); writing – review and editing (lead).

## FUNDING INFORMATION

This work was supported by the National Natural Science Foundation of China (No. 81503271 and No. 81573539), University Nursing Program for Young Scholars with Creative Talents in Heilongjiang Province (No. UNPYSCT‐2017217), and the Heilongjiang Touyan Innovation Team Program.

## CONFLICT OF INTEREST STATEMENT

The authors declare no conflicts of interest in this study.

## ETHICS STATEMENT

This study was approved by Institutional Animal Care and Use Committee of Heilongjiang University of Chinese Medicine.

## Supporting information


Table S1

Table S2

Table S3

Table S4

Table S5

Table S6

Table S7

Table S8

Table S9

Table S10

Table S11

## Data Availability

The data are available from the corresponding author on reasonable request.
